# Therapeutic Potential of Carnosine in Ischemia–Reperfusion Injury: A Preclinical Study in Muscle Tissue

**DOI:** 10.1155/crp/2496873

**Published:** 2025-08-26

**Authors:** Gokhan Erol, Hakan Kartal, Ertan Demirdaş, Gokhan Arslan, Tayfun Ozdem, Basak Yavuz

**Affiliations:** ^1^Gulhane Training and Research Hospital, Department of Cardiovascular Surgery, University of Health Sciences, Ankara, Turkey; ^2^Faculty of Medicine, Department of Histology and Embryology, Izmir Democracy University, Izmir, Turkey

**Keywords:** carnosine, ischemia–reperfusion, MDA, rats, TUNEL

## Abstract

**Background:** Ischemia–reperfusion (IR) injury, a process involving the disruption and subsequent restoration of blood flow, is a significant contributing factor to both cardiovascular diseases and broader tissue damage. Carnosine, a natural dipeptide notably abundant in muscle tissue and recognized for its antioxidant attributes, may offer protective benefits against the deleterious effects of IR injury.

**Methods:** A total of 24 rats were randomly allocated into four distinct groups: control, carnosine control, IR, and Carnosine + IR. The IR and Carnosine + IR groups underwent a simulated blood flow blockage lasting 120 min, followed by 120 min of reperfusion. Animals in the carnosine-treated groups received 250 mg/kg of carnosine via intraperitoneal injection prior to the experimental procedure. Muscle tissue samples were subsequently analyzed to quantify markers indicative of oxidative stress, inflammation, and cellular demise.

**Results:** Our findings demonstrated that, when compared to control groups, the IR group exhibited a significant elevation in key markers of oxidative stress (total oxidant status [TOS], Oxidative Stress Index [OSI]), inflammation (myeloperoxidase [MPO]), and cell death (TUNEL, Necrosis, Edema). Specifically, the IR group presented with a TOS of 8.72 ± 0.97 μmol/L, an OSI of 2.03 ± 0.18, and an MPO level of 75.93 ± 5.72 U/L, contrasting with control values of 4.23 ± 0.56 μmol/L, 1.01 ± 0.13, and 43.26 ± 5.7 U/L, respectively. Histopathological assessments corroborated these findings, revealing severe necrosis (2.50 ± 0.55), edema (2.00 ± 0.63), and notable inflammatory cell infiltration (2.67 ± 0.52) within the IR group. Furthermore, apoptosis (quantified by TUNEL assay) was significantly increased to 18.83 ± 1.47% in the IR group. Carnosine administration in the Carnosine + IR group led to a substantial reduction in all these adverse markers, bringing their levels closer to those observed in the control groups. For instance, in the Carnosine + IR group, TOS decreased to 5.63 ± 0.87 μmol/L, OSI to 1.24 ± 0.25, and MPO to 55.91 ± 3.45 U/L. Similarly, histopathological scores for necrosis, edema, and inflammatory cell infiltration were markedly lower in the Carnosine + IR group.

**Conclusion:** Our experimental findings strongly suggest that exogenously administered carnosine significantly reduces oxidative stress, suppresses inflammation, and attenuates cell death in skeletal muscle subjected to IR injury. These results highlight carnosine's promising therapeutic potential as a pharmacological agent for mitigating tissue damage in ischemic conditions.

## 1. Introduction

Ischemia–reperfusion (IR) injury represents a prevalent clinical challenge, stemming from conditions such as peripheral vascular trauma, compartment syndrome, or crush injuries, which can culminate in significant dysfunction within the extremities and various distant organs. Among the tissues in an extremity, skeletal muscle is particularly susceptible to ischemic damage. Given that muscle constitutes the primary tissue mass in these limbs, muscle injury is a central concern within the reperfusion syndrome. This detrimental process is characterized by a complex cascade of biological events, including oxidative stress, inflammation, and, ultimately, cellular demise, collectively leading to extensive tissue impairment and functional decline [1].

Carnosine (β-alanyl-L-histidine) is an endogenous dipeptide found across humans and diverse animal species. It participates in numerous physiological functions, such as antioxidant activity, membrane stabilization, and pH buffering [2]. Its distinct chemical configuration, notably encompassing an imidazole ring, enables it to directly neutralize reactive oxygen species (ROS) and reactive nitrogen species (RNS), chelate metal ions, and impede protein glycation and lipid peroxidation–processes integral to oxidative stress, a key component of IR injury [3, 4]. While carnosine has been the subject of considerable research, its complete spectrum of biological effects remains an area of ongoing investigation [3]. Prior investigations have demonstrated carnosine's roles as an antioxidant, a physiological buffer, an immune system modulator, and a neurotransmitter [3]. More specifically, carnosine has been identified as a modulator of nuclear factor erythroid 2-related factor 2 (Nrf2), a crucial regulator of antioxidant responses [5], and reportedly suppresses the production of proinflammatory cytokines like TNF-α and IL-6 [6, 7]. Carnosine is found in elevated concentrations within metabolically active tissues such as the brain, skeletal muscle, and heart muscle [8].

Within the scope of the current investigation, our objective was to ascertain the impact of carnosine on skeletal muscle tissue subjected to IR injury in a rat model involving the lower extremity.

## 2. Materials and Methods

### 2.1. Chemicals and Ethical Considerations

For this investigation, carnosine (Catalog Number: C9627) was obtained from Sigma-Aldrich, located in St. Louis, MO, USA. No financial or material contributions were accepted from external organizations or commercial entities for this specific study. All experimental procedures involving animal subjects strictly adhered to the International Standards for the Care and Use of Laboratory Animals. The research protocols were ethically approved by the Gazi University Animal Experiments Local Ethics Committee (Approval Code: G.U.ET-20.003) and were executed at the Gazi University Laboratory Animal Breeding and Experimental Researches Center (GUDAM). All supplementary chemicals and reagents utilized throughout the entirety of the study were of analytical grade.

### 2.2. Animal Housing and Grouping

A total of 24 male Wistar albino rats, whose body weights ranged from 250 to 350 g, were included in this research. The animals were housed in standard cages, with three rats per cage, within a controlled environmental setting. This environment was maintained at a temperature of 21°C–24°C and a relative humidity of 50% for a 7-day acclimatization period. A consistent 12 h light/12 h dark cycle was rigorously maintained. Throughout the entire experimental duration, all rats had unrestricted access to standard rodent chow and fresh drinking water. After the acclimatization phase, the animals were randomly assigned into four distinct experimental cohorts, each consisting of 6 rats: a Control group (C), a Carnosine Control group (C), an IR group, and a Carnosine + IR (C-IR) group.

### 2.3. Surgical Procedure and Experimental Design

Before commencing surgical intervention, a regimen of general anesthesia was administered, combining ketamine (at a dosage of 90 mg/kg) and xylazine (at 10 mg/kg). To proactively avert coagulation, all experimental groups were given an intravenous heparin bolus (100 IU/kg) 30 min prior to the surgical sequence. The abdominal area was meticulously prepared through shaving and subsequent antiseptic cleansing. Confirmation of adequate anesthetic depth was obtained before the surgical process was initiated. All surgical maneuvers were executed with rats positioned supine, under the continuous warmth of a heat lamp to ensure the maintenance of normothermia. A midline laparotomy was performed, and the intestinal structures were carefully displaced using moist gauze to expose the infrarenal abdominal aorta (AA). An atraumatic microvascular clamp was then affixed to the AA, serving to induce an ischemic state. The complete cessation of blood flow, indicative of ischemia, was visually corroborated by the immediate disappearance of distal aortic pulsation upon clamp application. After a 120-min ischemic interval, the microvascular clamp was detached from the AA, thereby initiating a 120-min reperfusion phase. Successful re-establishment of blood flow was confirmed by the observable return of pulsation in the distal aorta following the clamp's removal. For the Control group rats, a comparable duration (240 min) of laparotomy and abdominal aortic dissection was undertaken, yet without the induction of IR. To mitigate both heat and fluid loss throughout the IR period, the peritoneal cavity received saline instillation, and the abdominal incision was temporarily covered with moist gauze after the sequential clamping and unclamping of the AA [9].

## 3. Experimental Groups and Treatment Protocols

• Control Group (*n* = 6): After heparin administration, these rats underwent a median abdominal incision, which was then closed without inducing abdominal aortic ischemia. Animals were euthanized under general anesthesia 4 hours following the procedure.
• IR Group (*n* = 6): Subsequent to heparin administration, a median abdominal incision was performed on these rats; however, no carnosine injection was administered. An atraumatic microvascular clamp was then positioned on the AA, followed by 120 min of induced ischemia and a subsequent 120 min of reperfusion. These rats were then humanely euthanized while still under anesthesia.
• Carnosine Group (*n* = 6): Rats within this cohort received an intraperitoneal injection of carnosine (250 mg/kg) one hour before the initiation of any experimental procedure. Intravenous heparin was administered 30 min prior to the procedure, but no ischemic state was induced. A median abdominal incision was made before closure, and rats were humanely euthanized 4 h after the procedure, under general anesthesia. This specific dosage of carnosine was chosen based on previous research confirming its protective capacities, including notable antioxidant and anti-inflammatory attributes, in various preclinical models involving oxidative stress and IR injury, with no reported acute toxicity [6, 10]. The one-hour pretreatment interval was selected to facilitate adequate absorption and systemic distribution of carnosine within tissues prior to the ischemic insult, a timeframe consistent with comparable prophylactic intervention studies in animal models [6].
• Carnosine + Ischemia–Reperfusion (C + IR) Group (*n* = 6): These rats were administered an intraperitoneal injection of carnosine (250 mg/kg) one hour preceding the induction of ischemia. A median abdominal incision was performed 30 min postheparin administration, and a microvascular clamp was placed on the AA. Following a 120-min period of ischemia and a subsequent 120 min of reperfusion, these rats were humanely euthanized under anesthesia.

### 3.1. Tissue Homogenization

Hind limb tissue specimens were promptly collected and placed into sterile Eppendorf tubes and then immediately cryopreserved at −80°C until they could undergo biochemical analysis for total antioxidant status (TAS)/total oxidant status (TOS) and oxidative stress index (OSI). To prevent degradation, samples were rapidly weighed and flash-frozen. The frozen tissue was subsequently comminuted into a powder using liquid nitrogen within a prechilled porcelain mortar. One gram of this pulverized tissue was then homogenized in a precooled tube containing a 140 mM KCl solution, employing a 1:10 dilution ratio. To maintain a consistently low temperature and inhibit sample degradation, the homogenization tube was kept within an ice-filled glass beaker and agitated at 50 rpm for 2 minutes both preceding and following the homogenization process. The resulting homogenates were then transferred to fresh Eppendorf tubes, securely sealed, and subjected to centrifugation at 3000 rpm for a duration of 10 min. The supernatant was subsequently decanted into a new Eppendorf tube for the ensuing quantification of TOS and TAS [11].

### 3.2. TAS Measurement

The TAS levels within the samples were quantified using a fully automated Mindray BS300 analyzer, in conjunction with the specialized Relassay kit. The procedure involved an initial step where 300 μL of Reagent 1 (serving as the measurement buffer) and 18 μL of the sample were combined within a cuvette, followed by an initial absorbance reading at 660 nm after a 30-s interval. Subsequently, 45 μL of Reagent 2, which contained colored 2,2-azino-bis-3-ethylbenzothiazoline-6-sulfonic acid (ABTS), was introduced. Following a five-minute incubation period, a second absorbance measurement at 660 nm was performed. For calibration and standardization of the assay, a 1 mmol/L Trolox equivalent solution was utilized. Both the initial and final absorbance readings were performed in triplicate, and their respective average values were computed. The change in absorbance (ΔAbs) was derived by subtracting the first absorbance value (A1) from the second (A2). Final TAS levels were then calculated using the specific formula provided by the kit and expressed in units of mmol Trolox Eq/L (TAS = ∆AbsH_2_O − Abs sample/∆AbsH_2_O − Abs standard). This assay effectively measures the collective capacity of both enzymatic and nonenzymatic antioxidants to neutralize free radicals, thereby providing an indicator of the tissue's overall defense mechanism against oxidation.

### 3.3. TOS Measurement

TOS levels were also analyzed using a Mindray BS300 automated system in conjunction with the Relassay kit. The automated procedure commenced by dispensing 300 μL of Reagent 1 (the measuring buffer) and 45 μL of the sample into a cuvette. After mixing, an initial absorbance reading was taken at 530 nm following a 30-s interval. Subsequently, 15 μL of Reagent 2 (the pro-chromogenic solution) was introduced, mixed, and then incubated for 5 minutes before a second absorbance reading was acquired at 530 nm. For calibration, a standard solution provided by the kit, containing 10 μmol/L hydrogen peroxide (H_2_O_2_) equivalent/liter, was employed. Both sets of measurements were conducted in triplicate, and their respective average values were computed. The change in absorbance (ΔAbs) was determined by subtracting the first absorbance value (A1) from the second (A2). TOS levels were then calculated using the kit's specific formula and reported as mmol H_2_O_2_ Eq/L (TOS = ∆ Abs sample/∆ Abs standard × standard concentration 10 μmol/L). This assay furnishes a quantitative assessment of various oxidant molecules, thereby reflecting the cumulative oxidative burden present within the tissue.

### 3.4. OSI Calculation

The OSI was derived by establishing the ratio of the TOS to the TAS. Prior to this calculation, the TAS unit was converted to µmol/L. The final OSI value (expressed in arbitrary units) was then computed based on the formula: OSI = TOS (µmol H_2_O_2_ equivalent/L)/TAS (µmol Trolox equivalent/L) [12–14].

### 3.5. Myeloperoxidase (MPO) Measurement

MPO demonstrates a range of catalytic functionalities, primarily facilitating the production of hypochlorous acid (HClO) from hydrogen peroxide (H2O2) and chloride anions (Cl-). Additionally, MPO exhibits peroxidase activity, which involves the catalysis of substrate oxidation via H2O2. Both of these mechanisms are extensively employed to quantify MPO activity [15]. For the quantitative, colorimetric assessment of MPO activity in samples, two distinct Relassay kits were utilized: the Myeloperoxidase Chlorination Activity Assay Kit (Catalog Number: 6.0718.59.00.00) and the Myeloperoxidase Peroxidation Activity Assay Kit (Catalog Number: 6.0718.59.00.00). In the chlorination activity assay, MPO mediates the creation of hypochlorous acid, which then reacts with taurine to form taurine chloroamine. This taurine chloroamine subsequently interacts with the chromophore TNB, leading to the generation of colorless DTNB. One unit of MPO activity is operationally defined as the quantity of enzyme that hydrolyzes the substrate and produces taurine chloramine, resulting in the consumption of 1.0 µmole of TNB per minute. For the peroxidation activity assay, MPO catalyzes the conversion of *o*-dianisidine into a colored *o*-dianisidyl radical, using H_2_O_2_ as a co-substrate. The resultant rise in absorbance is monitored kinetically at 412 nm. Both assay kits are designed for manual use but can also be readily adapted for operation on automated analyzers.

### 3.6. Malondialdehyde (MDA) Measurement

The concentration of MDA, a widely recognized indicator of lipid peroxidation, was quantified through the application of the thiobarbituric acid (TBA) assay. In brief, tissue homogenates were combined with two volumes of chilled 10% (w/v) trichloroacetic acid (TCA) to facilitate protein precipitation. Following centrifugation, the resulting supernatant was incubated with an equivalent volume of 0.67% (w/v) TBA solution in a boiling water bath for a period of 10 min. The reaction was terminated by cooling, and the absorbance of the pink chromogen that formed was measured spectrophotometrically at 532 nm. The MDA concentration was then expressed in nanomoles per gram (nmol/g) of wet tissue, with its value determined by reference to a previously established standard curve.

### 3.7. Histopathological Analysis

Tissue specimens earmarked for histopathological examination underwent immediate fixation in a 10% neutral buffered formalin solution. Following a 48-h fixation period, the samples were subjected to standard histopathological processing protocols, culminating in their embedding within paraffin blocks. Five-micron-thick sections, prepared from these paraffin blocks, were then stained with hematoxylin–eosin (H&E). The resulting stained sections were independently evaluated and graded for the severity of necrosis, edema, and inflammatory cell infiltration, adhering to a predefined standardized scoring system (0: no injury, 1: mild injury, 2: moderate injury, 3: severe injury) [4, 16]. Mild injury (Score 1) was characterized by the presence of dispersed necrotic muscle fibers, minimal interstitial fluid accumulation (edema), and a sparse presence of infiltrating inflammatory cells. Moderate injury (Score 2) encompassed more extensive necrosis (impacting up to 25% of the visual field), noticeable fluid accumulation, and moderate aggregations of inflammatory cells. Severe injury (Score 3) exhibited widespread muscle fiber necrosis (exceeding 25%), significant intercellular edema, and a dense infiltration of inflammatory cells, notably including prominent polymorphonuclear leukocytes. The assessment of these prepared and stained sections was conducted using an Olympus CX43 microscope, which was outfitted with an integrated camera system.

### 3.8. Determination of Apoptosis by Terminal Deoxynucleotidyl Transferase dUTP Nick End Labeling (TUNEL) Method

Apoptotic cells were identified utilizing the TUNEL technique. This was accomplished by employing a commercially available TUNEL kit (ApopTag Plus Peroxidase In Situ Apoptosis Detection Kit, LOT: 2789455 Merc), after the standard processes of deparaffinization and rehydration of the tissue sections. To ascertain the apoptotic index (AI), five distinct, randomly selected microscopic fields from each tissue section were meticulously examined under 400x magnification. Cells that displayed brown or black staining were classified as TUNEL-positive, indicative of apoptosis. The AI was then calculated as the percentage of these TUNEL-positive cells relative to the total number of cells counted within each field, using the formula: AI = (Number of positive cells/Total number of cells counted) × 100.

### 3.9. Statistical Analysis

All gathered data were presented as the mean ± standard deviation. Statistical evaluations predominantly employed nonparametric tests, a choice necessitated by the non-normal distribution of the data and the relatively small sample size (*n* = 6 per group), which was confirmed through the Shapiro–Wilk test. For the presentation of data, descriptive statistics, including mean and standard deviation, were utilized. The normality of data distributions for measurements about necrosis, edema, inflammatory cell infiltration, TUNEL, TAS, TOS, OSI, MPO, and MDA across all study groups was specifically assessed using the Shapiro–Wilk test. Given the non-normal distributions observed for these variables and the consistent group size (*n* = 6), a nonparametric analytical approach was deemed appropriate for the study. Differences among the four experimental groups for all aforementioned measurements (necrosis, edema, inflammatory cell infiltration, TUNEL, TAS, TOS, OSI, MPO, and MDA) were analyzed via the Kruskal–Wallis test. Subsequent pairwise comparisons, conducted to pinpoint specific distinctions between groups, were performed using the Mann–Whitney *U* test. A *p* value of less than 0.05 (*p* < 0.05) was established as the criterion for statistical significance. All analytical procedures were executed using SPSS 22.0 software.

## 4. Results

This investigation was conducted to ascertain the protective effects of carnosine against muscle tissue injury induced by IR in rats. The analysis revealed statistically significant differences (*p* < 0.05) among the experimental groups across various measured parameters, thereby highlighting the potential therapeutic benefits associated with carnosine administration.

### 4.1. Enhanced Antioxidant Status

Regarding TAS, the Carnosine Control group exhibited significantly elevated levels when compared to all other cohorts (*p*=0.01) (Table 1). Specifically, the Carnosine Control group registered a TAS value of 0.54 ± 0.07 mmol/L, which was considerably higher than the Control (0.42 ± 0.05 mmol/L), IR (0.43 ± 0.04 mmol/L), and Carnosine + IR (0.46 ± 0.03 mmol/L) groups. This finding implies an initial augmentation in antioxidant capacity resulting from carnosine intervention.

### 4.2. Mitigation of Oxidative Stress

For TOS, both the IR and Carnosine + IR groups showed notably increased levels in comparison to the control and Carnosine Control groups (*p* < 0.05) (Table 1). However, a distinct reduction in TOS levels was observed in the Carnosine + IR group (5.63 ± 0.87 μmol/L) compared to the IR group alone (8.72 ± 0.97 μmol/L) (*p*=0.03). This outcome indicates a partial protective influence of carnosine against overall oxidative burden.

Concerning the OSI, the IR group presented a substantially higher OSI (2.03 ± 0.18) compared to all other experimental groups (*p*=0.01). While carnosine treatment did not fully normalize OSI values, it did induce a significant decrease in the index within the Carnosine + IR group (1.24 ± 0.25) when contrasted with the IR group (*p*=0.01). This suggests carnosine's potential to alleviate oxidative stress.

### 4.3. Reduced Inflammation

Regarding MPO levels, both the IR and Carnosine + IR groups demonstrated significantly elevated values compared to the Control and Carnosine Control groups (*p* < 0.05) (Table 1). However, the Carnosine + IR group exhibited markedly lower MPO levels (55.91 ± 3.45 U/L) in contrast to the IR group alone (75.93 ± 5.72 U/L) (*p*=0.01). This finding suggests that carnosine exerts a partial ameliorating effect on the inflammatory response in muscle tissue.

### 4.4. Attenuation of Cell Death

Consistent with the MPO findings, MDA levels were notably increased in the IR (0.46 ± 0.03 nmol/g) and Carnosine + IR (0.35 ± 0.03 nmol/g) groups when compared to the Control (0.22 ± 0.08 nmol/g) and Carnosine Control (0.26 ± 0.03 nmol/g) groups (*p* < 0.05) (Table 1). Nevertheless, the Carnosine + IR group displayed reduced MDA levels compared to the IR group alone. This outcome indicates a potential protective action of carnosine against lipid peroxidation and subsequent cellular damage.

### 4.5. Necrosis, Edema, and Inflammatory Cell Infiltration

**Figure 1:**
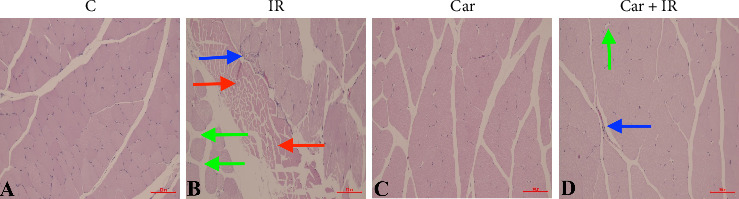
Histological sections stained with H-E from the experimental groups. Images (A–D) correspond to the control, ischemia–reperfusion, carnosine, and carnosine + ischemia–reperfusion groups, respectively. The photographs illustrate regions of necrosis (indicated by a red arrow), edema (indicated by a green arrow), and inflammatory cell infiltration (indicated by a blue arrow) (magnification × 200).

Histological analysis performed using H-E staining (Figure 1) revealed elevated levels of necrosis, edema, and inflammatory cell infiltration within the IR group, in distinct contrast to both the Control and Carnosine Control groups. These observations align with the increased MPO levels previously noted, further substantiating the presence of inflammation and tissue damage ensuing from IR (Table 2). Specifically, the IR group presented severe manifestations across all three parameters, indicative of significant tissue destruction and robust immune cell recruitment. Necrotic areas denote irreversible cellular demise, contributing to the compromised integrity of muscle tissue. Edema, characterized by interstitial fluid accumulation, impairs tissue perfusion and nutrient exchange, thereby intensifying the overall injury. Furthermore, inflammatory cell infiltration, particularly by neutrophils (as evidenced by MPO levels), exacerbates secondary tissue damage through the release of proteases and ROS.

### 4.6. TUNEL Assay

**Figure 2:**
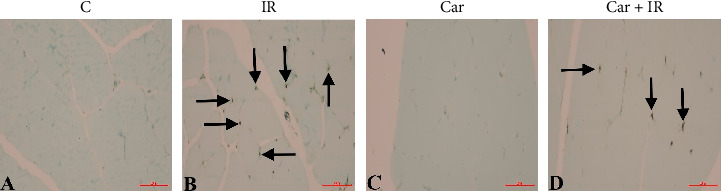
Photographs of sections stained with the TUNEL method to evaluate apoptosis in the experimental groups. The photographs display sections stained using the TUNEL method for the assessment of apoptosis across the experimental groups. Panels (A–D) correspond to the Control, Ischemia–Reperfusion, Carnosine, and Carnosine + Ischemia–Reperfusion groups, respectively. In these photographs, arrows specifically indicate TUNEL (+) apoptotic cells (magnification × 400).

TUNEL staining (Figure 2) confirmed significantly higher levels of apoptosis in the IR group (18.83 ± 1.47%) when compared to all other groups (*p* < 0.05). Although carnosine treatment did not lead to a complete normalization of TUNEL levels, it resulted in a notable decrease in the Carnosine + IR group (12.83 ± 1.47%) compared to the IR group alone. This suggests a potential protective effect of carnosine against cellular programmed cell death.

Overall, these findings collectively demonstrate that IR injury significantly exacerbates oxidative stress, inflammation, and cellular demise within muscle tissue. Pretreatment with carnosine effectively attenuates these adverse effects, thereby indicating its substantial therapeutic potential in mitigating IR-induced muscle damage.

## 5. Discussion

The current investigation provides compelling evidence that carnosine possesses substantial protective capabilities against skeletal muscle impairment resulting from IR injury. Our consistent findings demonstrate that carnosine administration effectively mitigates oxidative stress, reduces inflammation, and decreases cell death within muscle tissue subjected to IR injury.

Initially, our analysis of oxidative markers revealed a notable elevation in TAS in the Carnosine Control group, even under normal physiological conditions. This observation suggests that carnosine inherently enhances the body's antioxidant defenses. The mechanisms of many diseases involve the overactivation of free radical processes and a disruption of the organism's antioxidant protection systems [4], with IR injury being a prime example. While the human endogenous antioxidant response system rigorously controls reactive species to minimize cellular damage, the role of exogenous antioxidants is also crucial, as they have been shown to significantly impact this system [5]. Exogenous antioxidants, by collaborating with the endogenous system, provide a more advanced and effective defense against detrimental redox modulations [4]. More importantly, while IR alone led to significantly increase TOS and OSI, carnosine treatment in the Carnosine + IR group resulted in a substantial decrease in both parameters when compared to the IR group. These outcomes signify a direct ameliorating effect of carnosine on the oxidative burden and a beneficial shift toward antioxidant dominance in the tissue's redox balance, aligning with existing research on carnosine's potent antioxidant characteristics and its ability to scavenge free radicals.

Furthermore, our observations revealed significant decreases in MPO and MDA levels within the Carnosine + IR group when compared to the IR group. MPO and MDA serve as established markers for inflammation and lipid peroxidation, respectively, both of which are known to be considerably elevated during IR injury [6]. High levels of circulating MPO are typically associated with inflammatory processes and heightened oxidative stress [17]. MDA, being an end-product of lipid peroxidation, signifies increased free radical generation subsequent to reperfusion [18]. Prior investigations have documented an increase in MDA and MPO levels across all stages of IR, with a direct correlation between the duration of the reperfusion period and the severity of tissue injury [19]. Consequently, the quantification of MDA and MPO plays a critical role in assessing reperfusion injury. The observed decline in these markers within our study strongly supports carnosine's potent anti-inflammatory and antioxidative capabilities. These findings are consistent with existing research, indicating that prolonged reperfusion exacerbates tissue damage and elevates these inflammatory and injury markers. Our results imply that carnosine effectively curtails this detrimental progression, potentially by modulating the activation or infiltration of inflammatory cells, or by directly inhibiting MPO activity and associated proinflammatory pathways.

Histopathological assessments, including evaluations of necrosis, edema, and inflammatory cell infiltration, provided visual confirmation of carnosine's protective effects. H&E-stained sections unequivocally demonstrated a significant amelioration of these critical indicators of tissue injury within the Carnosine + IR group. This histological evidence further substantiates our biochemical findings and underscores carnosine's capacity to preserve muscle tissue integrity. These protective attributes may be attributed to carnosine's diverse mechanisms of action, such as its high concentration in inducible and long-lived tissues like skeletal muscle and brain [20], and its ability to modulate immune responses and stabilize cellular membranes, thereby restricting cellular damage and maintaining tissue architecture.

Our study additionally contributes to the existing understanding of carnosine's potential antiapoptotic properties. The scientific literature contains some conflicting data regarding carnosine's influence on apoptosis, with various studies suggesting it may either induce [10] or prevent [21] programmed cell death, depending on the specific experimental system and concentration utilized. Consequently, research into carnosine's antiapoptotic activity remains an active area of investigation [22]. Previous studies have also examined carnosine's role in human neutrophil function, demonstrating its capacity to increase interleukin-1β production and inhibit apoptosis, thereby suggesting its involvement in regulating the immune response within neutrophils [7]. In our current study, apoptotic cell counts in muscle tissues were assessed using the TUNEL method. Our findings indicate that carnosine effectively reduced the apoptosis induced by IR. We hypothesize that carnosine, which was also observed to decrease necrotic cells in eosin staining, exerts a cell-protective effect by inhibiting signaling pathways that lead to both apoptosis and necrosis.

The significant concentration of carnosine in metabolically active tissues with extended lifespans, such as skeletal muscle, is well-established. It is known that carnosine levels in muscle tissue decline with aging, concurrently with reductions in muscular endurance and strength. For instance, carnosine concentrations in muscle can decrease by up to 63% between the ages of 10 and 70, contributing to age-related loss of muscle mass and function [23]. Our findings align with antiaging research demonstrating carnosine's ability to delay senescence and prolong cell lifespan in cultured human diploid cells [24, 25], thereby emphasizing its cytoprotective and restorative potential across various oxidative injury scenarios. Based on our results, carnosine, a compound naturally present in muscle tissue, exhibits a protective effect against reperfusion injury in muscle, and exogenous administration further enhances this protective outcome.

## 6. Conclusion

In conclusion, our experimental findings strongly indicate that the exogenous administration of carnosine significantly reduces oxidative stress, suppresses inflammation, and mitigates cell death in skeletal muscle affected by IR injury. These results highlight carnosine's promising therapeutic utility as a pharmacological agent for alleviating tissue damage under ischemic conditions. Future research should focus on further elucidating the specific underlying mechanisms for each of these observed effects of carnosine on muscle tissue.

## 7. Limitations

It is important to acknowledge certain inherent limitations of this study. The relatively small sample size (*n* = 6 per group) and the use of a single carnosine dosage regimen may restrict the broad applicability and statistical robustness of our findings. Therefore, future research is warranted, incorporating larger sample sizes, varied dosing strategies to determine precise therapeutic ranges, and investigations into potential sex-related differences in response. Furthermore, while our study demonstrates beneficial outcomes, a more detailed exploration of the molecular and cellular mechanisms underpinning each of carnosine's protective actions (e.g., specific signaling pathways, alterations in gene expression) requires further in-depth investigation.

## Figures and Tables

**Table 1:** Evaluation of total antioxidant status (TAS), total oxidant status (TOS), oxidative stress index (OSI), myeloperoxidase (MPO), and malondialdehyde (MDA) levels across the different groups.

Measurement	Rats control [1]	Carnosine control [2]	Rats IR [3]	Carnosine + IR [8]	*p*	Difference
	*X* ± sd	*X* ± sd	*X* ± sd	*X* ± sd		
TAS (mmol/L)	0.42 ± 0.05	0.54 ± 0.07	0.43 ± 0.04	0.46 ± 0.03	0.01^∗^	1-2 (*p*=0.01)^∗^
						1–3 (*p*=0.93)
						1–4 (*p*=0.85)
						2-3 (*p*=0.01)^∗^
						2–4 (*p*=0.01)^∗^
						3-4 (*p*=0.18)

TOS (µmol/L)	4.23 ± 0.56	4.13 ± 1.76	8.72 ± 0.97	5.63 ± 0.87	0.01^∗^	1-2 (*p*=0.82)
						1–3 (*p*=0.01)^∗^
						1–4 (*p*=0.02)^∗^
						2-3 (*p*=0.01)^∗^
						2–4 (*p*=0.02)^∗^
						3-4 (*p*=0.03)^∗^

OSI	1.01 ± 0.13	0.76 ± 0.32	2.03 ± 0.18	1.24 ± 0.25	0.01^∗^	1-2 (*p*=0.34)
						1–3 (*p*=0.01)^∗^
						1–4 (*p*=0.11)
						2-3 (*p*=0.01)^∗^
						**2–4 ( ** **p**=0.05** )**
						3-4 (*p*=0.01)^∗^

MPO (U/L)	43.26 ± 5.7	44.78 ± 2.1	75.93 ± 5.72	55.91 ± 3.45	0.01^∗^	1-2 (*p*=0.98)
						1–3 (*p*=0.01)^∗^
						1–4 (*p*=0.01)^∗^
						2-3 (*p*=0.01)^∗^
						2–4 (*p*=0.01)^∗^
						3-4 (*p*=0.01)^∗^

MDA	0.22 ± 0.08	0.26 ± 0.03	0.46 ± 0.03	0.35 ± 0.03	0.01^∗^	1-2 (*p*=0.90)
						1–3 (*p*=0.01)^∗^
						1–4 (*p*=0.01)^∗^
						2-3 (*p*=0.01)^∗^
						2–4 (*p*=0.01)^∗^
						3-4 (*p*=0.01)^∗^

*Note:* The bold values in Table do not indicate statistical significance; all statistically significant differences correspond to *p* < 0.05. The asterisk (^∗^) indicates values with statistically significant differences (*p* < 0.05) compared to the relevant control or comparison group, as determined by Kruskal–Wallis and subsequent Mann–Whitney U tests.

**Table 2:** Assessment of necrosis, edema, inflammatory cell infiltration, and TUNEL levels across the groups.

Measurement	Rats control [1]	Carnosine control [2]	Rats IR [3]	Carnosine + IR [8]	*p*	Difference
	*X* ± sd	*X* ± sd	*X* ± sd	*X* ± sd		
Necrosis	0.33 ± 0.52	0.67 ± 0.52	2.50 ± 0.55	1.17 ± 0.41	0.01	1-2 (*p*=0.82)
						1–3 (*p*=0.01)^∗^
						1–4 (*p*=0.03)^∗^
						2-3 (*p*=0.01)^∗^
						2–4 (*p*=0.04)^∗^
						3-4 (*p*=0.01)^∗^

Edema	0.17 ± 0.41	0.33 ± 0.52	2.00 ± 0.63	0.83 ± 0.75	0.01	1-2 (*p*=0.99)
						1–3 (*p*=0.01)^∗^
						1–4 (*p*=0.01)^∗^
						2-3 (*p*=0.01)^∗^
						2–4 (*p*=0.02)^∗^
						3-4 (*p*=0.01)^∗^

Inflammatory cell infiltration	0.17 ± 0.41	0.50 ± 0.55	2.67 ± 0.52	1.33 ± 0.52	0.01	1-2 (*p*=0.88)
						1–3 (*p*=0.01)^∗^
						1–4 (*p*=0.01)^∗^
						2-3 (*p*=0.01)^∗^
						2–4 (*p*=0.01)^∗^
						3-4 (*p*=0.01)^∗^

TUNEL	2.50 ± 1.22	3.33 ± 0.52	18.83 ± 1.47	12.83 ± 1.47	0.01	1-2 (*p*=0.83)
						1–3 (*p*=0.01)^∗^
						1–4 (*p*=0.01)^∗^
						2-3 (*p*=0.01)^∗^
						2–4 (*p*=0.01)^∗^
						3-4 (*p*=0.01)^∗^

*Note:* The asterisk (^∗^) indicates values with statistically significant differences (*p* < 0.05) compared to the relevant control or comparison group, as determined by Kruskal–Wallis and subsequent Mann–Whitney U tests.

## Data Availability

The data that support the findings of this study are available from the corresponding author upon reasonable request.
